# Vectorial drivers of malaria transmission in Jabi Tehnan district, Amhara Regional State, Ethiopia

**DOI:** 10.1038/s41598-024-64436-3

**Published:** 2024-06-13

**Authors:** Aklilu K. Belay, Abebe Asale, Catherine L. Sole, Fiona Kinya, Abdullahi A. Yusuf, Baldwyn Torto, Clifford M. Mutero, David P. Tchouassi

**Affiliations:** 1 International Centre of Insect Physiology and Ecology, P.O. Box 30772-00100, Nairobi, Kenya; 2 International Centre of Insect Physiology and Ecology, P.O. Box 5689, Addis Ababa, Ethiopia; 3 Department of Zoology and Entomology, University of Pretoria, Private Bag X0028, Pretoria, South Africa; 4 School of Health Systems and Public Health, University of Pretoria, Private Bag X0028, Pretoria, South Africa

**Keywords:** Residual malaria transmission, Cryptic vectors, Outdoor biting, Malaria surveillance, Highland areas, Ecology, Ecology, Medical research

## Abstract

Among the factors affecting the effectiveness of malaria control is poor knowledge of the entomologic drivers of the disease. We investigated anopheline populations as part of a baseline study to implement house screening of windows and doors as a supplementary malaria control tool towards elimination in Jabi Tehnan district, Amhara Regional State of Ethiopia. The samples were surveyed monthly using CDC light traps between June 2020 and May 2021. Mosquito trap density (< 3 mosquitoes/trap) was low, however, with a high overall *Plasmodium* sporozoite rate (9%; indoor = 4.3%, outdoor = 13.1%) comprising *P. falciparum* (88.9%) and *P. vivax* (11.1%). *Anopheles gambiae* s.l., mostly *An. arabiensis*, comprised > 80% of total anopheline captures and contributed ~ 42% of *Plasmodium*-infected mosquitoes. On the other hand, morphologically scored *Anopheles funestus* s.l., constituting about 6% of anopheline collections, accounted for 50% of sporozoite-infected mosquitoes. Most of the infected *An. funestus* s.l. specimens (86.7%) were grouped with previously unknown or undescribed *Anopheles* species previously implicated as a cryptic malaria vector in the western Kenyan highlands, confirming its wider geographic distribution in eastern Africa. Other species with *Plasmodium* infection included *An. longipalpis* C, *An. theileri*, *An. demillioni,* and *An. nili.* Cumulatively, 77.8% of the infected mosquitoes occurred outdoors. These results suggest efficient malaria parasite transmission despite the low vector densities, which has implications for effective endpoint indicators to monitor malaria control progress. Additionally, the largely outdoor infection and discovery of previously unknown and cryptic vectors suggest an increased risk of residual malaria transmission and, thus, a constraint on effective malaria prevention and control.

## Introduction

Malaria remains a public health menace in Ethiopia^1^. While the disease in humans is caused by five different *Plasmodium* species globally, *Plasmodium falciparum* and *P. vivax* are the two major protozoan parasites responsible for the documented burden in Ethiopia^1,2^. Parasite transmission in the country is largely driven by *Anopheles arabiensis* as the primary vector while other species including *An. pharoensis*, *An. coustani* s.l., *An. nili,* and *An. funestus*, and *An. demeilloni* play minor roles^2^. Like most malaria-endemic countries worldwide, implementation of a suite of strategies over the past two decades (i.e., long-lasting insecticidal nets (LLINs), and indoor residual spraying (IRS), prompt case diagnosis and treatment) led to significant declines in malaria cases and consequent mortalities in most parts of Ethiopia. For instance, a 31% reduction in malaria cases and accompanying decline in deaths (58%) were observed between 2016 and 2020^3^.

Amidst the decline, compared to the instances in 2021, a 32% higher incidence was reported nationwide in 2022^4^. In addition, malaria transmission remains highly heterogenous across the country in different risk areas, partly influenced by climatic factors such as rainfall, altitude, and human population movements. A review of clinical data in health facilities in the Oromia Special Zone, Amhara Regional State of Ethiopia (2014–2019) revealed a 12.5% malaria parasite positivity rate with changes in the age-associated burden being highest in populations aged > 15 years^5^. Their findings highlight the need for active vector and parasite surveillance to track changes in malaria dynamics as interventional efforts are implemented^6^. As a critical component of vector control programs, surveillance includes measures such as assessing changes in vector composition and density, behaviour changes, pathogen infection rates and risk of transmission; and guiding timely decisions regarding interventions and allocation of resources efficiently.

Effective malaria control towards attainment of elimination, requires monitoring of critical entomological endpoints such as mosquito survival, host preference, species and infection status, and susceptibility to interventions^7,8^. Improved surveillance is essential to guide targeted or new strategies in response to changes in local transmission dynamics^6,9^. In Ethiopia, surveillance efforts have revealed emerging threats to malaria control including widespread pyrethroid resistance as in *An. arabiensis,* and likewise in the recently introduced vector *An. stephensi*^10,11^. In fact, this latter species is fast spreading in the country^10,12^ and already implicated in the spread of drug-resistant malaria parasites^13^. Recently, *An. pretoriensis* and *An. cinereus* were incriminated as novel vectors found to harbour the malaria parasite *P. falciparum* in the highlands of the Northwestern part of the country^14^.

Lately, house screening was evaluated as a supplementary intervention towards achieving malaria elimination in the Jabi Tehnan district, West Gojjam Zone, Amhara Regional State, Northwestern Ethiopia^15^. An important requirement for evaluating the efficacy of interventions entails characterization of the baseline malaria epidemiology including information on the driving vectors, genetic structure and associated bionomic features. Thus, the objective of the present study was to describe malaria entomologic risk factors pertaining to anopheline composition, abundance trends and *Plasmodium* infection rates. Additionally, we analysed the genetic diversity of anopheline populations found to harbour the malaria parasites.

## Materials and methods

### Study site

**Figure 1:**
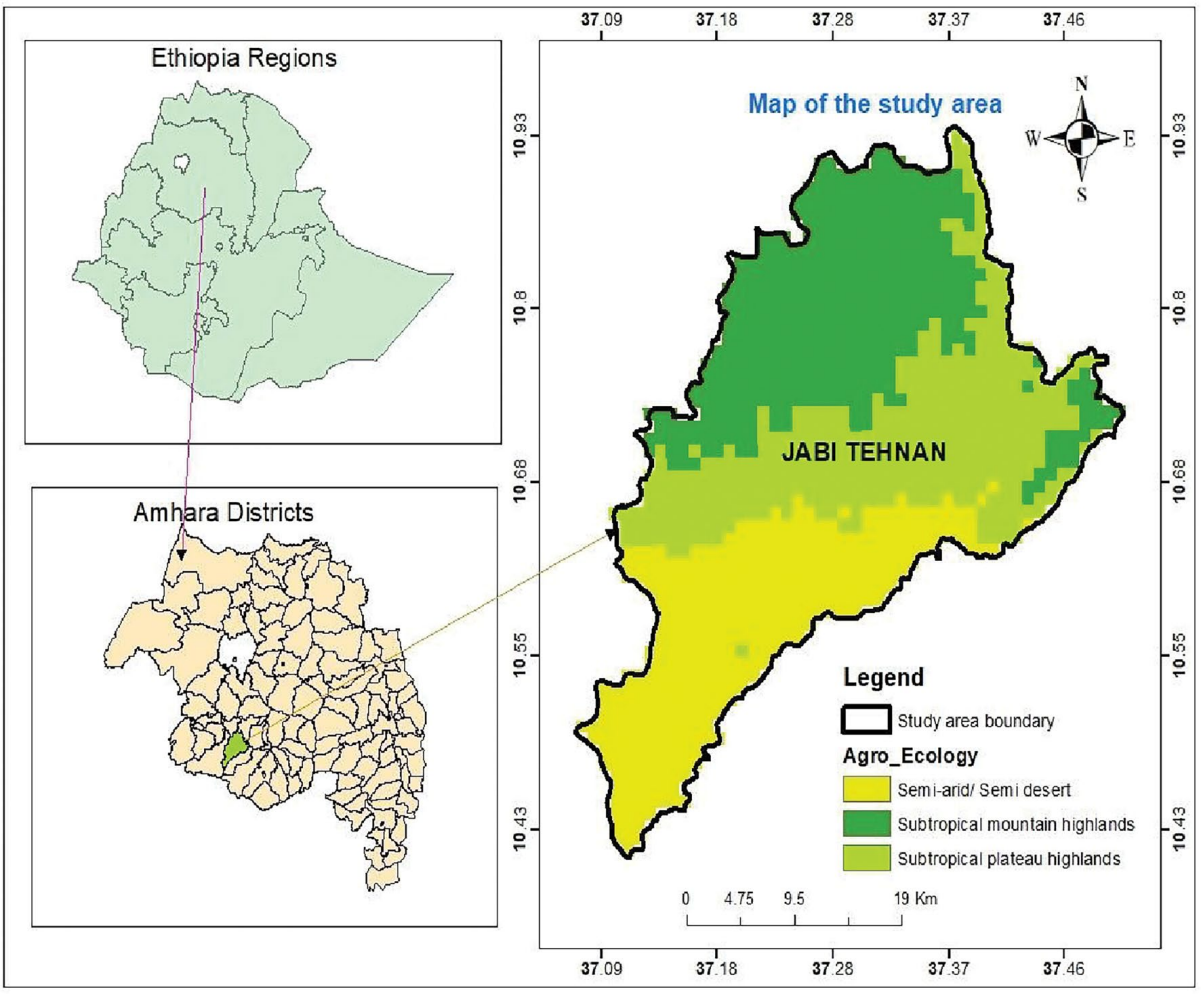
Map of the study area, Jabi Tehnan, a district in the West Gojjam Zone, Amhara regional state, Northwestern Ethiopia and its agroecological zones represented as follows, yellow = semi-arid/desert, green = subtropical dry mountain highlands and light green = subtropical plateau highlands.

This study was conducted in Jabi Tehnan district (37.074°–37.508°E; 10.405°–10.945°N), located in the West Gojjam Zone, Amhara Regional State of Northwestern Ethiopia (Fig. 1). The area has an altitude that ranges between 1300 and 2330 m above sea level (asl), and receives an annual precipitation of about 1356–1720 mm. Mean daily temperatures range from 15 to 20 °C. The district is endemic for malaria^16^ and encompasses 38 rural villages and 3 smaller towns with an estimated population of > 200,000 inhabitants residing in 45,827 rural and 7,927 urban households^17^. Malaria transmission is bi-modal with the peak occurring after the heavy rainy season (June to September) and another during the short rainy season (March to May). Recent data in the district indicate about 75% reduction in malaria cases between 2015 (24,427) and 2019 (6119)^18^.

### Study design, field mosquito sampling and identification

Surveys of adult host seeking anophelines were conducted in village clusters in three agro-ecological settings (June 2020–May 2021) with heterogeneous transmission and unknown entomological drivers. These settings include: (i) ‘subtropical dry mountainous highland’ (dry mountain) found > 2000 m asl, (ii) ‘subtropical plateau mid-highlands’ (plateau highlands) (1800–2000 m asl), and (iii) ‘subtropical semi-arid/semi-desert lowlands’ (semi-arid) (< 1800 m asl) (Fig. 1). We trapped mosquitoes using Centre for Disease Prevention and Control (CDC) light traps (Model 512, John W. Hock, Gainesville, FL, USA), deployed both indoors and outdoors in selected households each day. Each sampling month focused on four randomly selected households in a village representative of each agroecological setting. Two traps were placed in each household (1-indoor, 1-outdoor) making a total of eight traps set in a selected village per zone. Thus, a total of 24 randomly selected households were targeted across the agroecological area each sampling month. To ensure a spatial coverage, the same plan was enforced targeting different sets of randomly selected households. Traps were operated from 18:00 h to 06:00 h (local time) the following day. Mosquitoes captured were knocked down using chloroform, sorted and then identified morphologically to species level using published keys^19,20^. The physiological status of each female was classed as either blood-fed, gravid, half-gravid or unfed through observation of the abdomen^21^.

### DNA extraction

Extraction of genomic DNA (gDNA) from head/thorax from individual adult females was achieved using the ISOLATE II Genomic DNA Kit (Bioline, Meridian Bioscience, Germany) as per the protocol of the manufacturer. The DNA solution was used for malaria parasite detection via PCR (Polymerase Chain Reaction) assays as well as to identify sibling species in species complexes (described below).

### Mosquito screening for malaria parasite infection

A combination of methods was used for screening of *Plasmodium* infection in the samples. These included PCR-HRM (high resolution melting) analysis of the non-coding mitochondrial sequence (ncMS) followed by sequencing. Details of the PCR conditions are described in Kinya et al.^6^. Also, nested PCR targeting the 18S rRNA^22^ that detects and discriminates *Plasmodium* sporozoite of the species *P. falciparum*. *P. vivax*, *P. malariae* and *P. ovale*, was employed. The simian malaria parasite *P. knowlesi* is not known to infect humans in Africa. The first round of amplification in a 20 µL reaction volume comprised 10 µM of genus-specific primers rPLU-5 and rPLU-6, 4 µL of 2 × MyTaq Mix (Bioline, Meridian Bioscience, Germany), 0.2 U MyTaq polymerase, 10.58 µL of PCR water and ~ 20 ng of gDNA. The second round of amplification entailed same constituents except template being 4 µL of the first PCR product and species-specific primer pairs each in separate PCRs (rFAL1 + rFAL2, rVIV1 + rVIV2, rMAL1 + rMAL2, and rOVAL1 + rOVAL2)^23^. In both first and second round amplification, the cycling conditions were 5 min at 95 °C; 25 cycles (first round, 30 cycles for second round) of 30 s at 94 °C, 2 min at 58 °C and 2 min at 72 °C and final extension for 5 min at 72 °C. The nested PCR products were separated on a 2% agarose gel to identify the *Plasmodium* species. The third method specifically detected sporozoite of *P. falciparum* by amplifying the single copy gene glutathione reductase (PfGR) using the primers PfGR_F 5′-GCTGCCTCAGTTCATGATATTT-3′ and PfGR_R 5′-CTTATCCCTTCTCTCTACCAACAG-3′^24^. Each PCR reaction contained, 4 µL of 2 × MyTaq Mix, 0.2 U MyTaq polymerase, 11.6 µL of PCR water, 20 ng of gDNA and 10 µM of each primer. The thermal profile used for PCR was: 5 min at 95 °C, 30 cycles of 30 s at 94 °C, 30 s at 60 °C and 30 s at 72 °C and final extension for 5 min at 72 °C. Amplicons were verified on 2% agarose gel electrophoresis against a 100 bp DNA HyperLadder (Bioline, Meridian Bioscience, Tennessee, USA). Negative (uninfected *An. arabiensis* DNA) and positive control (DNA from each of the *Plasmodium* species from National Institute for Biological Standards and Control (NIBSC; London, UK) were included in PCR runs.

### Identification of the sibling species of *An. gambiae* complex

The HRM-PCR protocol by Zianni et al.^25^ that distinguishes *An. arabiensis* from *An. gambiae* was used, the former species being the main primary vector in the *An. gambiae* s.l. in Ethiopia^2^. The PCR used primers that targeted the ribosomal internal transcribed spacer subunit-2 (ITS2) region. The DNA amplification with the Solis Biodyne kit (Solis BioDyne, Tartu, Estonia), in a 10 μL reaction mix contained 2 μl of 5X Hot Firepol EvaGreen HRM Mix, 0.5 μM of each primer, ~ 20 ng of DNA template and PCR water. Positive (DNA of lab-reared *An. arabiensis* maintained at the Insectary Unit, *icipe* Duduville Campus), Nairobi and negative (PCR grade water) controls were included. The thermal conditions entailed an initial denaturation (1 min at 95 °C) followed by 40 cycles of denaturation (95 °C for 30 s), annealing (57 °C for 30 s), and extension (72 °C for 45 s) with a further extension at 72 °C for 7 min. Generated HRM profiles were analysed using HRM analysis tools in the RGQ software (Qiagen). *Plasmodium* sporozoite-positive *An. gambiae* s.l. specimens were similarly processed to identify the sibling species as described above.

### Molecular analysis of morphologically scored *An. funestus* mosquitoes

This was limited only to morphologically scored *An. funestus* group specimens that tested positive for *Plasmodium* sporozoite infection. They were analysed by PCR amplification of the internal transcribed spacer 2 (ITS2) region of the ribosomal DNA (rDNA)^26^ using established protocols^6^. This genetic marker has been widely used to discriminate closely related anophelines and other mosquito species with abundant sequences available in public databases (e.g., GenBank). Briefly, PCRs performed in a 15 μL reaction volume comprised 0.5 μM of the forward and reverse primers, 3 μL of 5X HOT FIREPol Blend Master Mix (Solis BioDyne, Estonia) and ~ 20 ng of DNA template. Thermal cycling conditions were 95 °C for 15 min followed by 40 cycles of denaturation at 95 °C for 30 s, annealing at 46 °C for 30 s and extension at 72 °C for 40 s followed by final extension at 72 °C for 10 min. Amplicons were confirmed by gel electrophoresis and the PCR products similarly purified and sequenced as explained earlier.

### Sequence and phylogenetic analyses

Mosquito sequences were viewed and edited in Chromas, embedded in MEGA v.6.0^27^ and queried in GenBank using Basic Local Alignment Search Tool (BLastn). Multiple sequence alignments of the resulting contiguous sequences were performed using MUSCLE in MEGA v.6.0 with default parameters. A neighbor-joining (NJ) tree was inferred for ITS2 sequences of infected *An. funestus* mosquitoes using the p-distance method with nodal support for different groupings evaluated through 1000 bootstrap replications. Indels were excluded from analysis.

### Ethical approval

Review of the study protocol and ethical approval were given by the Regional Public Health Research Ethics Review Committee /RERC/ (Ref. No: H/R/T/T/D/5/3) from Amhara Public Health Research Institute, Ethiopia. The research activity was started by providing information about the whole objective of the study to the community and consensus obtained from household owners for the project related activities done with the directions given by local stakeholders.

### Statistical analyses

Relative abundance was used to estimate the composition of the anopheline mosquitoes indoors and outdoors. Daily counts of female mosquito/trap for *An. gambiae* s.l. were compared between the agro-ecological zones using generalised linear models (GLM) with negative binomial error structure based on best-fit model residuals. Replicate trap catches were pooled for the village clusters in each agro-ecological area viz*:* plateau highlands, dry mountains and semi-arid. The main predictor variables were agro-ecology and location (indoor/outdoor). The mean catches/trap/night was computed for each of the four species that comprised > 90% of the total catches: *An. gambiae* s.l., *An. funestus* s.l., *An. coustani* s.l. and *An. squamosus*. Pair-wise mean comparisons in the abundance of these species indoors versus outdoors for each agroecology was ensured using the package *emmeans* with a p-value adjustment equivalent to Tukey test. The *P. falciparum* sporozoite infection rates (*Pf*sp) were expressed as the number of positive specimens of the total number examined for each species and the proportion compared between indoors and outdoors using Pearson χ^2^ test. All analysis was implemented in R v. 4.2.1^28^ including GLMs with the MASS package, and results considered significant at p ≤ 0.05.

## Results

### Anopheline mosquito composition

**Figure 2:**
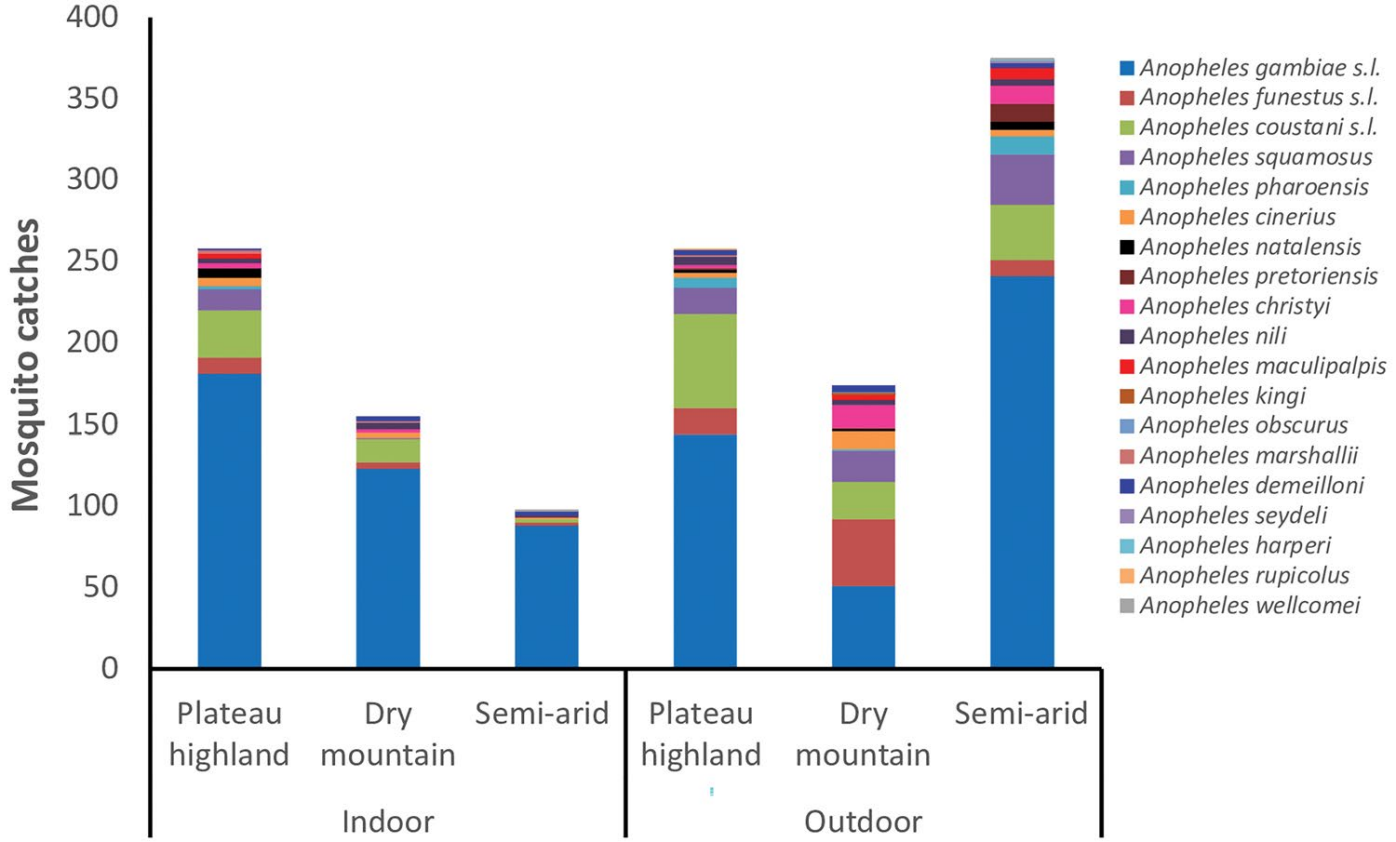
Anopheline mosquito composition based on morphology, collected in Jabi Tehnan district, Amhara Regional State, Northwestern Ethiopia. The number of trap nights (n) were indoors: plateau highland = 69, dry mountain = 61, semi-arid = 64; outdoors: plateau highland = 82, dry mountain = 70, semi-arid = 90.

A total trapping effort of 436 trap nights (194 indoors, 242 outdoors) yielded 1318 anopheline females comprising 19 species. The differences in the replicates were attributed to malfunctioned traps that resulted in zero catches and were excluded from the experiment. The species trapped and classified based on morphology were *Anopheles gambiae* s.l., *An. funestus* s.l., *An. coustani* s.l., *An. squamosus*, *An. pharoensis*, *An. cinereus*, *An. natalensis*, *An. pretoriensis*, *An. christyi*, *An. nili*, *An. maculipalpis*, *An. marshalli*, *An. demeilloni*, *An. rupicolus*, *An. wellcomei*, *An. kingi*, *An. obscurus*, *An. seydeli*, and *An. harperi* (Fig. 2).

The species occurrences were non-uniform across the different agro-ecological zones. *An. gambiae* s.l. was relatively predominant accounting for 62.8% of the total anopheline collections. This was followed by *An. coustani* s.l. (12.1%), *An. funestus* s.l. (6.3%) and *An. squamosus* (6.1%). Sparse occurrence of the other anophelines was observed with individual proportions ranging from 0.08 to 2.4% of the total anopheline catches.

Abundance trends were compared for *An. gambiae* s.l. and found not to differ by agroecologic areas (χ^2^_2,433_ = 318.10, p = 0.20) nor between indoors and outdoors (χ^2^_1,432_ = 317.20, p = 0.35). In contrast, captures of *An. funestus* s.l. mosquitoes varied by agroecology (χ^2^_2,433_ = 136.08, p = 0.005) being significantly higher both in dry mountain (Z value = 2.89, p = 0.004) and plateau highland (Z value 1.98, p = 0.048) than semi-arid. Also, the species were significantly encountered outdoors than indoors (Z value = 2.90, p = 0.004).

### Variation in mosquito densities

**Figure 3:**
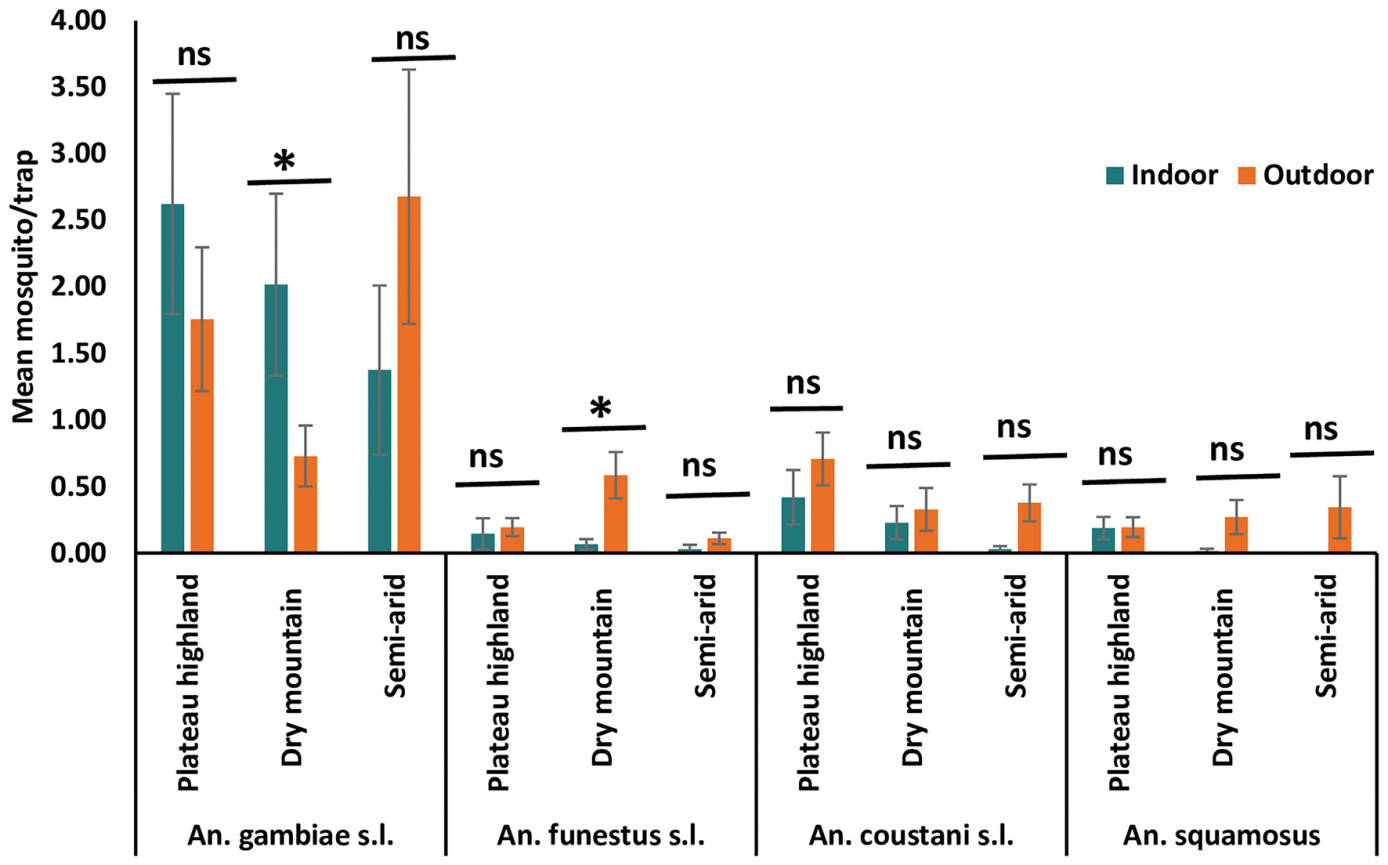
Mean captures (± se) of dominant anopheline species encountered in Jabi Tehnan district, Amhara Regional State, Northwestern Ethiopia. Comparisons are made between indoor and outdoor captures in each agroecology zone for each species, * significant difference at p < 0.05; ns, non-significant difference; se = standard error.

Mosquito trap densities were generally low (< 3 mosquitoes/trap) and varied between the three agro-ecological zones and indoors versus outdoors for certain species. This was compared for the four dominant species (*An. gambiae* s.l., *An. coustani* s.l., *An. funestus* s.l., and *An. squamosus* (comprised ~ 90% of total anopheline collections). Mean *An. gambiae* s.l. densities ranged from 1.38 to 2.68/trap-night and differed indoors and outdoors in the dry mountain highland areas. The generally low *An. funestus* s.l. densities (< 1/trap-night) were not significantly different between indoors and outdoors except in the dry mountain highland (Fig. 3). There was no significant difference in captures of *An. coustani* s.l. and *An. squamosus* between indoor and outdoor locations in any of the agroecological setting, although there was a tendency for more captures outdoors.

### Physiological status of mosquitoes

The mosquitoes were classified as blood-fed, gravid, half-gravid or unfed. Three hundred and one (301), 80 and 18 mosquitoes were blood-fed, gravid and half-gravid, respectively. This translated into a cumulative engorged (blood-fed + gravid + half-gravid) rate of 30.3% (399/1318) of the trap catches. Overall engorged rates were 37.7 and 27.7% for *An. gambiae* s.l. and *An. funestus* s.l., respectively. Higher rates were evident for other species, but they occurred in very low levels (Table 1).

**Table 1:** Summary of engorged (blood-fed, gravid and half gravid) anophelines of the total catches.

Mosquito species	Indoor No. engorged (rate, total catches)	Outdoor No. engorged (rate, total catches)	Total No. engorged (rate, total catches)
*Anopheles gambiae* s.l. *	161 (41.1%, 392)	151 (34.6%, 436)	312 (37.7%, 828)
*Anopheles funestus* s.l. *	3 (18.8%, 16)	20 (29.9%, 67)	23 (27.7%, 83)
*Anopheles coustani* s.l. *	3 (6.7%, 45)	13 (11.3%, 115)	16 (10%, 160)
*Anopheles squamosus*	2 (14.3%, 14)	5 (7.6%, 66)	7 (8.75%, 80)
*Anopheles pharoensis*	1 (50%, 2)	2 (11.1%, 18)	3 (15%, 20)
*Anopheles cinereus*	3 (5.6%, 9)	1 (33.3%, 18)	4 (14.8%, 27)
*Anopheles natalensis*	2 (33.3%, 6)	1 (12.5%, 8)	3 (21.4%, 14)
*Anopheles pretoriensis*	1 (0%, 1)	4 (30.8%, 13)	5 (35.7%, 14)
*Anopheles christyi*	3 (60%, 5)	6 (22.2%, 27)	9 (28.1%, 32)
Anopheles nili *	3 (42.9, 7)	2 (16.7, 12)	5 (26.3%, 19)
*Anopheles maculipalpis*	1 (33.3%, 3)	4 (40%, 10)	5 (38.5%, 13)
*Anopheles kingi*	0	1 (0%, 1)	0 (0.0%, 1)
*Anopheles obscurus*	0	1 (0%, 1)	0 (0.0%, 1)
*Anopheles marshallii*	1 (100%, 1)	0 (0%, 3)	1 (25.0%, 4)
*Anopheles demeilloni*	1 (14.3%, 7)	2 (20%, 10)	3 (17.7%, 17)
*Anopheles seydeli*	0	1 (0%, 1)	0 (0.0%, 1)
*Anopheles harperi*	0	1 (0%, 1)	0 (0.0%, 1)
Anopheles rupicolus *	0	1 (100%, 1)	1 (100%, 1)
*Anopheles wellcomei*	1 (100%, 1)	1 (100%, 1)	2 (100%, 2)

* Species found infected with *Plasmodium * parasites.

### *Plasmodium* sporozoite infection rates in anopheline mosquitoes

Head/thorax of only engorged cohorts was tested for *Plasmodium* infection, of which five anopheline species tested positive: *An. gambiae* s.l., *An. funestus* s.l., *An. coustani* s.l., *An. nili,* and *An. rupicolus* (Table 2). Engorged cohort has had biting encounters at least once with a host (including humans) and more likely to be infected. *Plasmodium* infection prevalence in *An. gambiae* s.l. (all found to be *An. arabiensis*) was 4.8% (15/312) and higher outdoors than indoors but not significantly different (9/151 vs 6/161; χ^2^ = 0.43, df = 1, p = 0.51). Of the infection in *An. arabiensis*, *P. falciparum* was predominant (12/15) followed by *P. vivax* (3/15) (Table 2). A high proportion of *An. funestus* s.l. tested positive for *P. falciparum* (18/23, 78.3%) and infection was encountered across the agro-ecologic areas. Higher numbers of infectious *An. funestus* s.l. mosquitoes occurred outdoors compared to indoors but with comparable rates (16/20 vs 2/3; Table 2). One specimen each of *An. coustani* s.l., and *An. rupicolus* was infected with *P. falciparum* encountered outdoors in the plateau highland area. Also, one *An. nili* trapped outdoor tested positive for *P. vivax* in the semi-arid zone. Overall, infection prevalence was significantly higher outdoors relative to indoors (28/213 vs 8/186; χ^2^ = 8.42, df = 1, p = 0.004). In fact, 77.8% of the infectious mosquitoes were captured outdoors.

**Table 2:** *Plasmodium* sporozoite infection by species in the three agro-ecological areas in Jabi Tehnan district in Amhara Regional State, Northwestern Ethiopia. Numbers positive of total tested in brackets are indicated for each species.

	Plateau highlands		Dry mountain		Semi-arid		Total	
Mosquito species	Indoor	Outdoor	Indoor	Outdoor	Indoor	Outdoor	Indoor	Outdoor
*An. gambiae* s.l	3(65) (2Pf, 1Pv)	3(26) (2Pf, 1Pv)	2(54)	1(6)	1(42)	5(119) (4Pf,1Pv)	6(161)	9(151)
*An. funestus* s.l.*	1(1) (1Pf)	1(3) (1Pf)	0	12(13) (13Pf)	1(2) (1Pf)	3(4) (3Pf)	2(3)	16(20)
*An. coustani* s.l	0(2)	1(7)	0(1)	0	0	0(6)	0(3)	1(13)
*An. squamosus*	0(2)	0(1)	0	0	0	0(4)	2	5
*An. pharoensis*	0(1)	0(1)	0	0		0(1)	1	2
*An. cinereus*	0	0	0(2)	0	0(1)	0(1)	3	1
*An. natalensis*	0(2)	0	0	0	0	0(1)	2	1
*An. pretoriensis*	0	0	0	0	0(1)	0(4)	1	4
*An. christyi*	0	0(1)	0(3)	0	0	0(5)	3	6
*An. nili*	0	0	0(3)	0	0	1(2) (Pv)	3	1(2)
*An. maculipalpis*	0(1)	0(1)	0	0	0	0(3)	1	4
*An. marshallii*	0(1)	0	0	0	0	0	1	0
*An. demeilloni*	0(1)	0(2)	0	0	0	0	1	2
*An. rupicolus*	0	1(1) (Pf)	0	0	0	0	0	1(1)
*An. wellcomei*	0	0	0	0	0(1)	0(1)	1	1
Total	4(76)	6(43)	2(63)	13(19)	2(47)	9(151)	8(186)	28(213)

Values indicate number infected (total analysed), composition of the parasites: Pf = *Plasmodium falciparum*, Pv = *Plasmodium vivax*; * morphologically identified only pending molecular analysis. All infected *An. gambiae* s.l. was identified as *An. arabiensis*, Species found infected with *Plasmodium* parasites are in bold font; *morphologically scored before molecular analysis.

### Molecular analysis of mosquitoes

The 52 *An. gambiae* s.l. samples including *Plasmodium* infected and non-infected, were found to be *An. arabiensis*.

**Figure 4:**
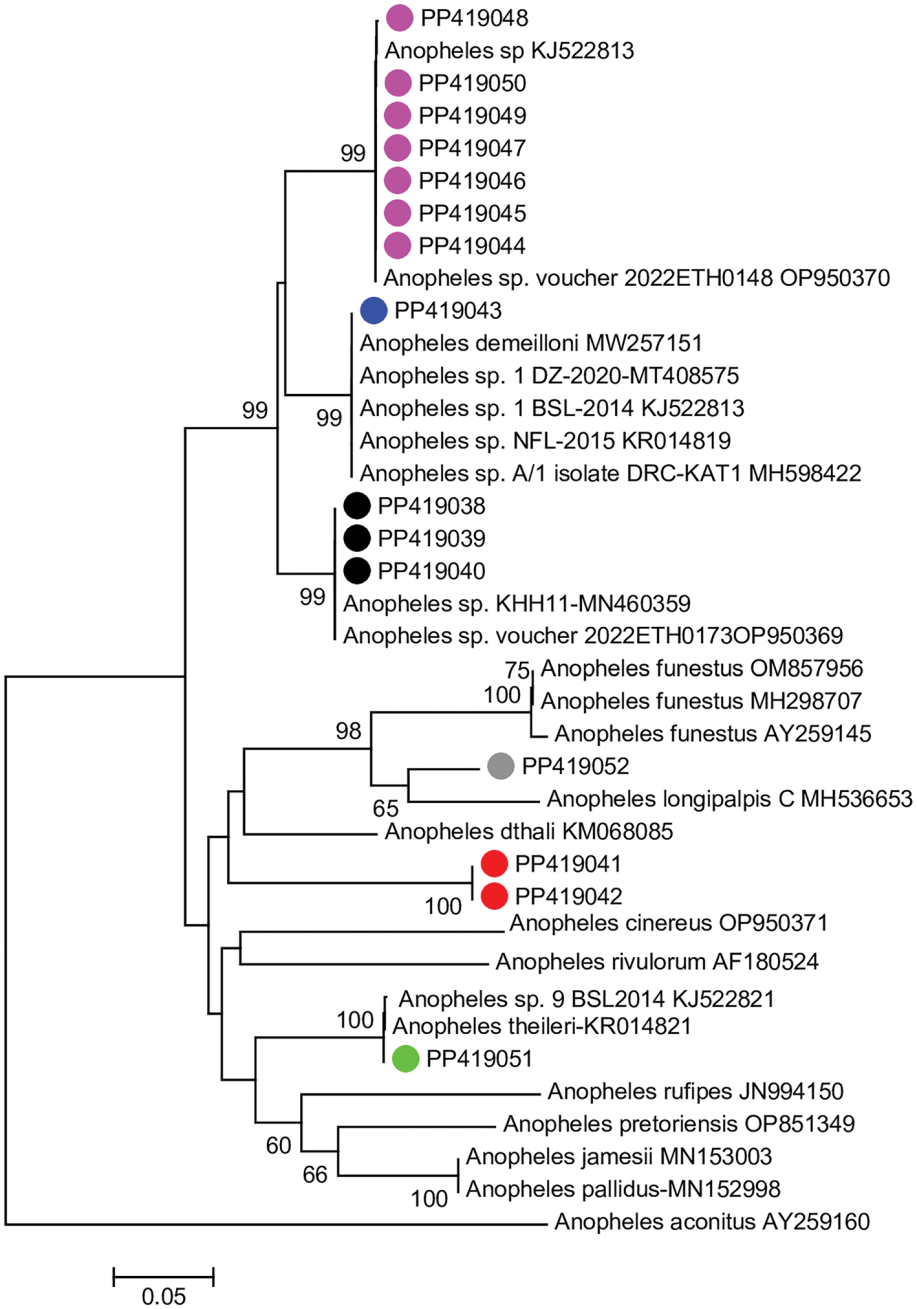
Neighbour-joining tree for sequences of mosquitoes (259–496 bp) morphologically scored as *An. funestus* group infected with *P. falciparum* sporozoites. Bootstrap support values are indicated at relevant nodes. Filled circles are sequences generated in this study from *P. falciparum* infected specimens; the novel cryptic species undescribed but previously reported in literature (pink/black), clustering with described species (blue/grey/green) or unreported species (red). Sequences of specimens harbouring *Plasmodium* sporozoite infection were deposited in GenBank (PP419038- PP419052).

To confirm the species identity of the *An. funestus* s.l. infected specimens, PCR of the ITS2 region and then sequencing and phylogenetic analyses were performed. Phylogenetic analysis of the successfully generated sequences (13/16) based on NJ trees showed the samples clustered in diverse clades that were well supported (Bootstrap (BS) values > 99%). Of the samples, one clustered with *An. longipalpis* C; one with *An. theileri*, 3 with a previously reported by uncharacterised species in Ethiopia (accession number OP950369), 1 with *An. demeilloni*, and the majority (n = 7) with yet-to-be described species recently detected in Ethiopia (accession number OP950370) and previously reported in Kenya as a malaria vector (accession no. KJ522813). Two positive specimens did not cluster with any known species (Fig. 4).

## Discussion

This study investigated and established baseline entomological determinants of malaria in Jabi Tehnan district in view of implementation of house screening for malaria control. The findings of the study depict an overall low density of anophelines (estimated by trap catches), but unexpected high infection rates in *An. arabiensis* and mosquitoes morphologically scored as belonging to the *An. funestus* group. The data on *An. arabiensis* is consistent with its primary vectoring role in the country^1,2,29^. Few parasite detections were associated with *An. coustani* and *An. nili* previously implicated as secondary vectors in parts of the country and elsewhere in Africa^30–32^. However, the association of wild *An. rupicolus* with the malaria parasite represents novel data for the country. Further, the high infection rate for the supposed *An. funestus* mosquitoes were unexpected which were found to not be any of the commonly known sibling species in the Funestus group. Instead, the results based on sequencing and phylogeny showed the specimens grouped in diverse well supported clades indicative of presence of different species. Two issues arise from the observed data. First is the likely case of misidentification. Some of the sequenced samples clustered with referenced samples of the species *An. theileri*, and *An. demeilloni*. Infection in *An. demeilloni* supports literature^1,2^ and its known role as secondary malaria vector in Ethiopia while infection in *An. theileri* represents a novel malaria parasite relationship in the country. Second is the presence of cryptic species, novel or previously reported that cannot be unravelled without molecular analysis^33,34^. Most of the infected specimens grouped with yet-to-be described *Anopheles* species previously implicated as a cryptic malaria vector in western Kenya highlands (accession no. KJ522813). This species was also recently reported in Ethiopia (accession number OP950370); however, we provide new data about its infection with *P. falciparum* in Ethiopia and its potential wider geographic distribution in eastern Africa. Likewise, *An. longipalpis* C incriminated in the dryland ecosystem of Kenya^6,9^ was observed for the first time harbouring *Plasmodium* parasite infection in Ethiopia. Together, the findings suggest possible evolution and shifting vectorial drivers of malaria transmission and importance of novel and perhaps cryptic vectors, in the malaria epidemiologic landscape as previously reported in East and southern Africa^6,32–34^. Whether this trend is a consequence of existing control measures requires close monitoring.

The high proportion of infected mosquitoes could indicate efficient transmission despite low overall mosquito densities. This was particularly obvious for the morphologically scored *An. funestus* mosquitoes which could be highly competent vectors and contributing to residual malaria transmission (RMT), i.e., persistence of malaria transmission following the implementation in time and space of a widely effective malaria programme^35^. This is particularly relevant given that a higher proportion of these mosquitoes occurred outdoors including those infected with *Plasmodium* parasites. The finding is indicative of outdoor malaria transmission that is less susceptible to traditional vector control methods (i.e., IRS, LLINs), and an important contributor to RMT^6,35^. Previous studies have argued for consideration of infection status over abundance or occurrence as a better risk indicator of dominance of a species in residual malaria^6,35^. While densities of *An. arabiensis* exceeded those of the morphologically scored *An. funestus* by higher order of magnitude (16:1 ratio), the latter still contributed more than 50% of malaria infections. The results have implications for effective endpoint indicators to monitor control progress; the proportion of infectious mosquitoes than routine monitoring of adult mosquito densities could represent the primary, most direct indicator of programme performance.

The extremely low densities yet high mosquito infectiousness remains paradoxical. Whether related to the competence of the populations as malaria is being controlled (i.e., hyper-endemic regions becoming meso-endemic) remains unknown. It has been postulated that efficiency of malaria parasite transmission (human-mosquito) increases as malaria is being controlled, likely attributed to human host factors relating to higher host gametocyte density^36^. Mosquito factors related to competence cannot be discounted if these species exhibit high survival abilities; these require further assessments. On the other hand, these mosquitoes could be poorly captured as adults in CDC light traps known to bias certain anopheline species^37^ likely explaining the observed low densities. Inclusion of human landing collections and animal or odour-baited tools should be explored in future studies^37^.

The uncovering of several cryptic species mostly undescribed via PCR and phylogeny highlights the limitation of morphologic identification. The high infection rates of these species suggest a substantial contribution to sustaining malaria in the study areas. As the threat of invasive species like *An. stephensi*^13^ gains traction, the role played by cryptic species in malaria transmission cannot be overlooked. Increasingly, reports of novel as well as cryptic vectors have been observed in malarious areas in Kenya and southern Africa^6,32–34^, indicating a growing menace that requires consideration. In the present study, only a subset of infected cohorts was processed for identification by PCR; thus, the species richness could even be much higher. Genetic characterisation of the vector populations using a suite of markers will aid in revealing the species richness and diversity in the studied ecologies and discriminate between vector and non-vector species that can mislead vector control strategies. A substantial proportion of *Plasmodium* infected mosquitoes were sampled outdoors (> 80%) representing a significant challenge beyond the reach of LLINs which are the primary commodities for malaria transmission control indoors. Thus, for malaria elimination to be achieved, outdoor biting fractions of anopheline vectors must also be effectively tackled^6^. Furthermore, despite the presence of bednets in households, mosquitoes were still captured indoors some of which were infected with malaria parasites. The factors which underlie this observation are unclear but could be associated with humans (e.g. net use patterns) or mosquitoes (e.g., insecticide resistance), that require further investigations.

## Conclusions

The results from this study show that in the Jabi Tehnan district of northwestern Ethiopia, there was an overall low densities of *Anopheles* mosquitoes evident in the low trap catches, both indoors and outdoors. While *An. arabiensis* was the predominant vector, undescribed but previously reported or unknown species seem to support transmission on account of their high infection rate yet low abundance. Uncovering of these species, highlights the need to integrate molecular techniques into routine malaria entomological surveillance. The presence of highly infectious mosquitoes is indicative of efficient transmission despite low densities and has implications for effective endpoint indicators to monitor malaria control progress. Most of the infectious mosquitoes occurred outdoors (~ 80%) raising the urgent need to tackle such fractions out of reach of existing interventions mainly deployed indoors. The present data provide strong evidence of possible associated changes in the vectorial landscape. Thus, monitoring for changing and perhaps novel threats should be a prerequisite as malaria control towards elimination is being pursued.

## Data Availability

Sequences of mosquito specimens harbouring *Plasmodium* sporozoite infection were deposited in GenBank (PP419038- PP419052).
